# Decrease ventilator-associated pneumonia by bundle care in cardiac surgery intensive care unit

**DOI:** 10.1186/2047-2994-4-S1-P241

**Published:** 2015-06-16

**Authors:** CT Hung, MH Hsieh, P-W Yang, S-W Wu, C-Y Lin, Y-H Chen

**Affiliations:** 1 Department of Infection Control, Department of Internal Medicine, Kaohsiung Medical University Hospital, Kaohsiung Medical University, Kaohsiung, Taiwan, Province of China; 2 Division of Infectious Diseases, Department of Internal Medicine, Kaohsiung Medical University Hospital, Kaohsiung Medical University, Kaohsiung, Taiwan, Province of China

## Introduction

Ventilator-associated pneumonia (VAP) is major healthcare-associated infections in intensive care unit (ICU), which increases length of stay, medical cost and mortality.

## Objectives

The infection density of cardiac surgery ICU in a tertiary teaching hospital in southern Taiwan was 4‰ during January 2011 to December 2012 which is higher than 2‰ in the other ICUs. In order to decrease the incidence of VAP, bundle care was applied in this ICU.

## Methods

After observing nursing care procedures in 24 intubated patients, some measurements were inappropriate, including mouth care once per day, odor smell in patient’s mouth, poor compliance of hand hygiene before and after patient contact, head elevation less than 30 to 45 degrees, no regular evaluation of extubation daily. Therefore, bundle care of VAP with following options was applied, including evaluation of oral hygiene every eight hours, oral hygiene with toothbrush and 2% chlorhexidine-containing fluid every hour or every eight hours based on individual difference, audit of hand hygiene, reminding symbol for head elevation at bedside, evaluation the necessary of extubation by physician, nurse practitioner and respiratory therapist daily.

## Results

After leading in bundle care and interprofessional practice, the infection density decreased from 4‰ during January 2011 to December 2012 to 0‰ during January 2013 to August 2014. The infection density remain zero for twenty months.

## Conclusion

VAP is common healthcare associated infection in ICU and leads to unexpected outcomes in patient care. All the medical staffs should place importance on the concept of bundle care to decrease the incidence of VAP.

## Disclosure of interest

None declared.

## References

[B1] Mohamed KAE Compliance with VAP bundle implementation and its effectiveness on surgical and medical sub-population in adult ICU Egypt. J. Chest Dis. Tuberc 2014 63 1 9 14 10.1016/j.ejcdt.2013.10.019

[B2] Lerma F Álvarez García M Sánchez Lorente L Gordo F Añón JM Álvarez J Palomar M García R Arias S Vázquez-Calatayud M Guidelines for the prevention of ventilator-associated pneumonia and their implementation The Spanish｀Zero-VAP´bundle. Med Intensiva 2014 38 4 226 236 10.1016/j.medin.2013.12.007 24594437

